# Do Benign Tumors Become Malignant?
*Do tumors of the breast, which may be classed as benign, ever become, by metaplasia, malignant; and if so what tumors are subject to that change?


**Published:** 1889-02

**Authors:** John B. Harvie

**Affiliations:** Physician to Troy, N. Y., Hospital.


ARTICLE III.

### DO BENIGN TUMORS BECOME MALIGNANT?*

BY JOHN B. HARVIE, M. D.

—Physician to Troy, N. Y., Hospital.—

[Read before the Troy Medical Association, Dec. 4th, 1888.]

There is no doubt but that the different growths which are found in other structures and parts of the body are capable of affecting and producing similar results in the breast. At first sight it would seem probable that a benign and unmalignant formation would never result in anything more than what it originally was. That a formation or neoplasm would remain and continue as it primarily began and never transform or change into anything else. When we have the poison of measles sown in the system we expect to find measles resulting from said contamination and not diphtheria, consequently if we have a simple non-malignant tumor of the breast, "or any other part of the body for that matter," we do not naturally look for the subsequent development of something which is essentially different in character, microscopical appearance and result as regards the patient.

Benign tumors of the breast have, until recently, been brought under one general class and named adenomata. Recent observers have, however, dissected out the subject fairly well, and have placed each species under a head of its own, according to the microscopical appearance and tissue of which it is composed. To the practical surgeon, however, a diagnosis is not always very easily arrived at. To distinguish between malignant and non-malignant growths, even in a situation so favorable for manipulation as in the breast, is a problem which occasionally taxes the

* Do tumors of the breast, which may be classed as benign, ever become, by metaplasia, malignant; and if so what tumors are subject to that change?

ingenuity of the most experienced, and it will be found impossible to avoid error at times. It may be laid down as a rule, however, that the nearer the structure of a growth approaches the normal physiological tissue the more probable is it that such a formation is non-malignant, and the wider the channel the greater the possibility of malignancy.

In regard to the development of neoplasms whether they are of epithelial or connective tissue origin the question arises must they originate and increase by proliferation of the tissue which they represent, or can a cell of one series give origin to one essentially different, or may the result of multiplication be modified by the presence of cells of different systems on the same principle as if we plant certain kinds of vines in the same bed, such as cucumbers and citron we shall have resulting a fruit which is altogether unlike either one or the other.

The metamorphosis of the connective tissue corpuscles by what is known as epithelial infection or inoculation has been regarded by some as a very probable theory. How this infection might arise and what might cause it is as mysterious as the transformation which takes place in the fruit.

In regard to the origin of tumors the theory which stands out most prominently, and the one most generally accepted, is that of local origin. The most recent theory, that of Cohnheim, demands an accumulation of dormant embryonal cells as such a cause. Cohnheim supports this view by experiments, in which he transplants fœtal tissue into a mature animal with the result of excessive development of that particular tissue. Same practised after birth gives a negative result. Growth possesses for its active element cells and cells are propagated only by segmentation or pre-existing cells. Tumors have failed to be propagated by inoculation. Experiments have been tried time and again on individuals and animals without showing any result. Cohnheim thereupon returns to this theory that the morbid growth must spring from embryonal cells, and that as ovum, by segmentation, gives origin to different varieties of normal tissues, different in function and structure, so we may have produced abnormal varieties of tissue, with like divergent manifestation of function. It is further assumed that the elements from which a tumor arises are of an indifferent character, and that the result of the growth, what its character, etc., may be, is influenced in some way by the condition and circumstances at the time.

What part predisposition takes in favoring the development of simple neoplasms of the mammary gland is probably disputable. Family history, however, plays so important a role in the predisposition to the development of other affections that it seems as though it must be accepted as an important factor. The married state and the physiological transformation which takes place in the breast during lactation and pregnancy offer very slight, if any, contributing cause. Lactation, however, in predisposing to inflammatory and sometimes suppurative changes in the breast, may be followed by areas of circumscribed hardness, which may consist of cicatricial connective tissue holding in its meshes more or less glandular structure. Such a mass might, however, be classed with such as result from injury.

It seems almost probable that growths which are inclined to be innocent in character are, as a rule, confined to the connective tissue, and those which are malignant to the glandular portion of the breast. The growth and development of innocent neoplasms takes place within a capsule and does not involve the surrounding tissue; that of malignant formations the very opposite.

In considering the subject of why benign tumors of the breast are liable to become malignant by metaplasia, I should say that the lipomata and certain cysts are recognized as being uniformly benign, and that certain other growths, which are regarded as innocent, may, under certain circumstances; take on malignant action. What the occasion is which excites the intruder to change his coat and practically become a horse of another color is still a mystery.

Benign growths sometimes assume very great dimensions, but never affect any alteration in surrounding tissue except that of displacement of parts and atrophy through pressure. They are essentially local in their development.

Sometimes tumors, by their peculiar behaviour, answer all the requirements of a malignant growth, while structurally they retain the proper arrangement to entitle them to a position within the harmless fold. As to what tumors may become malignant by metaplasia I should regard those known as fibroma, a fibrous tumor, myxoma or mucous tumor, and adenoma, a glandular tumor, as those which are most likely to take on cancerous action. That form known as chondroma, a cartilaginous tumor, bring very rarely, if ever so.

All tumors are subject to degeneration, decay and death. They may inflame, suppurate and die, resulting in a mass of chronic inflammatory tissue, penetrated by sinuses from all points and absolutely refusing to heal. And it is an indisputable clinical fact that those growths which are earliest in reaching maturity are the first to show signs of decay, and it is further shown by experience that the tumors which attain maturity most rapidly, even though they be ever so benign at the outset, are more prone to undergo a cancerous degeneration. The slower the growth the more likely will it be to remain benign.

If a tumor be purely of a fibrous character, its growth will inevitably be slow, and the possibility of any malignancy may almost positively be disregarded, but if such a growth should be the seat of vegetations or fibro-cystic in character then it increases in dimensions much more rapidly and indeed is sometimes a strong rival of carcinoma itself.

The disposition of fibro-cystic growths to take on malignant action must not be overlooked. It is possible that a neoplasm might remain innocent throughout its entire structure for many years and become the subject of cancerous deposit. Erichsen reports such a case. The tumor had existed more than twenty years before removal having commenced at the age of twenty-eight, but after extirpation cancerous matter was found at the bottom of some of the cysts, which, as the constitution was uncontaminated, was doubtless of recent formation, and the surface of the fungus was epitheliomatous.

The neoplasm known as myxoma or mucous tumor appears so seldom that its appearance is regarded almost as a curiosity. Its growth is somewhat rapid and is disposed to recur after removal. On account of the limited number of these neoplasms which have been recorded and the imperfect history of the cases, it is not known what percentage, if any, assumed malignant action.

The adenomata are also very rare growths. This species is probably more prone to undergo cystic degeneration than any other form of tumor. It is also more liable to recur after removal than any one of the simple neoplasms. Its progress is usually very chronic but occasionally assumes great rapidity of growth, thus almost simulating malignant disease. Although recurrence after removal in this form of neoplasm is quite great, still infection of neighboring organs seems to be very exceptional. When fatality occurs it seems to have been super-induced altogether on account of the local lesion or repeated operations and exhaustion in consequence rather than through any constitutional infection. Gross lays considerable stress on the liability of this form of tumor to become carcinomatous and considers the epithelium of the acini as the starting point.

Indurations of the breast in consequence of pre-existing mastitis are supposed to be a fruitful source for the development of malignant disease. Although these cicatrical masses may remain benign during the period of usefulness of the breast, still, when the age for the development of carcinoma arrives, and especially it any cachexia exists, the foundation of cancer is apt to be laid. Since the fact has been admitted that some forms of benign growths may assume malignant action it would stand almost to reason that any simple neoplasm which is liable to degenerate or take on irritative action may, providing the idiosyncrasy exists, become cancerous.—*Montreal Medical Journal.*

